# A multivariate statistical approach to predict COVID‐19 count data with epidemiological interpretation and uncertainty quantification

**DOI:** 10.1002/sim.9129

**Published:** 2021-08-10

**Authors:** Francesco Bartolucci, Fulvia Pennoni, Antonietta Mira

**Affiliations:** ^1^ Department of Economics University of Perugia Perugia Italy; ^2^ Department of Statistics and Quantitative Methods University of Milano‐Bicocca Milan Italy; ^3^ Faculty of Economics Università della Svizzera italiana (CH) Lugano Italy; ^4^ University of Insubria Varese Italy

**Keywords:** Dirichlet‐multinomial distribution, epidemic modeling, model diagnostics, multinomial distribution, pandemic predictions, reproduction number

## Abstract

For the analysis of COVID‐19 pandemic data, we propose Bayesian multinomial and Dirichlet‐multinomial autoregressive models for time‐series of counts of patients in mutually exclusive and exhaustive observational categories, defined according to the severity of the patient status and the required treatment. Categories include hospitalized in regular wards (H) and in intensive care units (ICU), together with deceased (D) and recovered (R). These models explicitly formulate assumptions on the transition probabilities between these categories across time, thanks to a flexible formulation based on parameters that a priori follow normal distributions, possibly truncated to incorporate specific hypotheses having an epidemiological interpretation. The posterior distribution of model parameters and the transition matrices are estimated by a Markov chain Monte Carlo algorithm that also provides predictions and allows us to compute the reproduction number $R_{t}$. All estimates and predictions are endowed with an accuracy measure obtained thanks to the Bayesian approach. We present results concerning data collected during the first wave of the pandemic in Italy and Lombardy and study the effect of nonpharmaceutical interventions. Suitable discrepancy measures defined to check and compare models show that the Dirichlet‐multinomial model has an adequate fit and provides good predictive performance in particular for H and ICU patients.

## 1 INTRODUCTION

We introduce Bayesian multinomial and Dirichlet‐multinomial statistical autoregressive models for the observed time series of COVID‐19 count data. We also design a Markov chain Monte Carlo (MCMC) simulation algorithm for parameter inference. The model based on a Dirichlet‐multinomial distribution is able to account for *overdispersion* and provides stable predictions, especially of the number of patients who need hospitalization and those who require intensive care. These predictions can support decision makers in designing better informed emergency management plans (see, among others, Reference 1), both at the beginning of the outbreak, when scaling up health‐care capacity is crucial to save lives, and in the later phase of the epidemic, to better plan the timing to return to usual capacity in each hospital ward.

Predictions should be trusted with care because the official data may present biases due to the observational nature and the delays of the collection process. For example with reference to Italy, it sometimes happens that some data collected over a period of several days are officialized in a single day, causing spikes in the time series. Furthermore, this reporting delay is not constant over time but rather emerges more prominently during the emergency phase. In this often biased observational context, an advantage of our proposal is that it automatically smooths spikes in the data that can be identified and later investigated to understand if they are due to reporting errors or other causes such as the resurgence of the pandemic, in which case a warning signal should be issued.

Besides timing related issues, as suggested by Roda et al^2^ prediction is very difficult when there is a lack of reliable data sources. One of the main problems, especially in Italy, is related to the fact that swabs to identify COVID‐19 virus infections have been dispensed only to those showing symptoms and having been in contact with a person who tested positive. This was the protocol at the beginning but it was changed a few times during the emergency period and these modifications also caused reporting biases. Therefore, due to nonrandomization of the tested people, lack of testing kits and, even more importantly, due to scarcity of labs accredited to process the swabs, as suggested by Roda et al^2^ “the entire iceberg represents the total infected population and the tip of the iceberg above the sea surface represents the case data.” This phenomenon is called hidden epidemic and caused a critical care crisis in Italy as well as in other countries.^3^

A series of univariate models such as the Poisson^4^ and models based on a negative binomial distribution,^5^, ^6^, ^7^ as well as the generalized logistic growth model,^8^ have been proposed for single time series of counts, especially to analyze Italian data. These models are often formulated with a temporal trend^9^ through polynomials and splines. Differently from the univariate models outlined above, the model we propose explicitly considers that the count for a certain category at a certain time occasion is the sum of transitions from the same and other categories that these individuals occupied at the previous time occasion. In other words, our model directly formulates assumptions on the sequence of contingency tables of the “transition frequencies” between two consecutive time occasions. This assumption directly induces the distribution of the total counts that, differently from the unobservable transition frequencies, are directly observed and should result as column totals of the contingency tables. The advantage of our multivariate modeling framework is in terms of stability and precision, leveraging on the fact that some of the counts on the variables included in the model are inherently less prone to measurement errors. These errors may arise for many reasons such as because people in the population who were infected by the virus remain asymptomatic during the first days of the infection. We also have to consider the undercounting of deaths caused by social isolation and other factors as detailed in Reference 10, as well as due to delays in the reporting schedule.

The proposed approach may be seen as an extension of that proposed in Reference 11 for 2×2 contingency tables. Moreover, it is related to the SEIR (Susceptible‐Exposed‐Infected‐Recovered) epidemiological model.^12^ Indeed, we explicitly model the reduction of the number of susceptible individuals, the virus transmission rate, the transfer rate from exposed to infected, the diagnosis, and the recovery rate. We note that according to the Italian regulations during the period of time we consider, the category of susceptible also includes asymptomatic cases. This is due to the health policy measures in place in Italy during the first wave of the pandemic when only individuals with symptoms were tested. Indeed, asymptomatic and pauci‐symptomatic cases are not reported among the “positive cases.” We also stress that the deceased category includes both people who died because of COVID‐19 as well as with COVID‐19. A subsequent analysis of the mortality cards revealed that 89% of the deaths are directly attributable to COVID‐19.*We can furthermore estimate the time evolution of the epidemic reproduction number together with credible bounds.

We cast our proposal in a Bayesian framework^13^, ^14^, ^15^ because it allows us to incorporate expert prior information that, when data are lacking, helps in regularizing the likelihood function, and allows for predictions at the very beginning of the pandemic period. Priors can also be informed from data available in countries where the epidemic started earlier, like data from Hubei, where the first cases were reported on 22 January 2020, approximately 10 days earlier than in Italy. By this time, in the Hubei area, more than 5800 cases were already present.†

The modeling strategy is flexible and proceeds in steps of increasing complexity. Our proposal is conceived to provide a model that can explore the available data and thus is first estimated with noninformative priors. Then, to account for epidemiological hypotheses, we introduce truncated priors enforced by imposing bounds to the admitted values of the odds of transition across categories. Finally we also account for public health non‐pharmaceutical interventions (NPI) enforced to reduce the spread of the epidemic, thus causing changes to the time series of reported confirmed cases. This is achieved by introducing dummies^16^ at certain time points to account for the effect of NPI. It is therefore important to retain the capacity to fit increasingly complex models. We also provide an estimate of the reproduction number $R_{t}$ following the method described in Reference 17, where the authors assume that the “serial interval” has a Gamma distribution with certain parameter values.

The multinomial and Dirichlet‐multinomial autoregressive models may be considered as stochastic processes following a first‐order Markov chain conditional on the latent disease status.^18^ In our formulation these models include absorbing states, as that of deceased patients. For each category, including that of the susceptible individuals not previously ill, recovered, and deceased, we apply Bayesian inference^14^ to estimate the persistence in each category, the transition probabilities between categories across time, and also the associated uncertainty of the estimates. Note that the assumption of first‐order dependence is typically formulated in the literature on time‐series categorical data. The motivation is that most of the relevant information to explain the counts at a given time occasion is contained in the counts at the previous occasion, and we consider this as plausible also in our context. In principle, this assumption could be relaxed by allowing for a higher‐order dependence. However, this type of extension would be very complex to implement and also for this reason we choose to retain a first‐order dependence.

For model estimation we adopt an MCMC algorithm^19^ that simulates parameter values from their posterior distribution. The algorithm is based on a unified data augmented scheme^20^ and comprises two iteratively repeated steps: the first step is based on sampling tables of transition frequencies using the technique described in References 21, 22; the second draws new values of the model parameters on the basis of moves based on a Metropolis‐Hastings (MH) acceptance rule.^23^, ^24^ Using a large number of iteratively generated MCMC draws, we obtain the estimated joint posterior distribution of parameters that is then summarized by marginal posterior averages and prediction intervals. To diagnose possible violations of the model assumptions and compare the performance of alternative models we use a suitable discrepancy measure and compute posterior predictive *p*‐values.^25^, ^26^, ^27^

The remainder of the article is organized as follows. In Section 2 we describe the proposed models. In Section 3 we illustrate the estimation of the model parameters, of the reproduction number together with predictions of various interesting quantities, and derive some discrepancy measures for model checking and comparisons. In Section 4 we show the results of the models estimated with the Italian data available during the first wave of the pandemic; we also report on the results obtained with data coming from the Lombardy region where the spread of the virus began in Italy. In Section 5 we provide some concluding remarks. In the Appendix some additional details are presented.

## 2 PROPOSED APPROACH

Data consist of counts, over *T* time occasions, for *K* disjoint and exhaustive categories that will be jointly modeled: $y_{tk},\;t\in 𝒯,k\in 𝒦$, where 𝒯={1,…,T} and 𝒦={1,…,K}. For each time occasion, these observed frequencies are collected in the vectors $y_{t}={\left(y_{t1},\ldots ,y_{tK}\right)}^{\prime }$, t∈𝒯. We assume, for simplicity, that the total population size is fixed over time, namely, $\sum_{k\in 𝒦}y_{tk}=N$ for all *t*. The corresponding random vectors are denoted by $Y_{t}$ and have elements $Y_{tk}$ that satisfy the same constraint on the sum over *k*. In the application referred to official data provided at the national level on the COVID‐19 pandemic, individuals are classified in K=6 ordered (in terms of their severity) categories: susceptible not previously ill (S), recovered (R), positive cases in quarantine (Q), hospitalized (H), intensive care (ICU), and deceased (D); for each of these categories we observe the frequency on a daily basis. The “now positive” (NP) category is obtained as the sum of individuals in the Q, H, and ICU categories.

### 2.1 Model assumptions

We consider the counts for the first time occasion, t=1, as given, and, in formulating an autoregressive model for the vector $Y_{t}$, we assume that every element $Y_{tk}$ is the column total of a contingency table having row totals equal to the elements $Y_{t-1,k}$ of $Y_{t-1}$ for t>1. In more detail, let $X_{tjk}$ represent one of the frequencies in this contingency table, a random variable corresponding to the number of individuals that at occasion t−1 are in category *j* and at occasion *t* move to category *k*. In symbols we have that $Y_{tk}=\sum_{j\in 𝒦}X_{tjk}$, for all *k*, are the column totals and $Y_{t-1,j}=\sum_{k\in 𝒦}X_{tjk}$, for all *j*, are the row totals. These column and row sums are the only observable variables since they are the only publicly provided counts. This structure is clarified in Table 1, where zero values are inserted to denote that the corresponding random variables are equal to zero with probability one (structural zeros). This is because state *D* is absorbing (zeros in the last row) and because we assume that, once infected, patients are not susceptible anymore (zeros in the first column).

**TABLE 1:** Data structure: $Y_{tk}$ denotes the observed count at occasion *t* of category *k* for each of the K=6 categories for the COVID‐19 application; $X_{tjk}$ denotes the number of transitions from category *j* to category *k* at time *t*

	S	R	Q	H	ICU	D	Total
**S**	$X_{t11}$	$X_{t12}$	$X_{t13}$	$X_{t14}$	$X_{t15}$	$X_{t16}$	$Y_{t-1,1}$
**R**	0	$X_{t22}$	$X_{t23}$	$X_{t24}$	$X_{t25}$	$X_{t26}$	$Y_{t-1,2}$
**Q**	0	$X_{t32}$	$X_{t33}$	$X_{t34}$	$X_{t35}$	$X_{t36}$	$Y_{t-1,3}$
**H**	0	$X_{t42}$	$X_{t43}$	$X_{t44}$	$X_{t45}$	$X_{t46}$	$Y_{t-1,4}$
**ICU**	0	$X_{t52}$	$X_{t53}$	$X_{t54}$	$X_{t55}$	$X_{t56}$	$Y_{t-1,5}$
**D**	0	0	0	0	0	$X_{t66}$	$Y_{t-1,6}$
**Total**	$Y_{t1}$	$Y_{t2}$	$Y_{t3}$	$Y_{t4}$	$Y_{t5}$	$Y_{t6}$	*N*

The unobserved random variables $X_{tjk}$ are collected in the vectors $X_{tj}={\left(X_{tj1},\ldots ,X_{tjK}\right)}^{\prime }$, j∈𝒦, $t\in {𝒯}^{\prime }$, where ${𝒯}^{\prime }=\{2,\ldots ,T\}$, and are here named “transition frequencies.” For instance, in our application on COVID‐19 illustrated in the sequel, where we consider six categories, $X_{t35}$ corresponds to the number of individuals that moved from category Q (number 3) at time t−1 into category ICU (number 5) at occasion *t*.

It is natural to assume that every vector $X_{tj}$, given $Y_{t-1}$, follows a multinomial distribution with size $y_{t-1,j}$ and specific vector of “transition probabilities” $p_{tj}={\left(p_{tj1},\ldots ,p_{tjK}\right)}^{\prime }$ with elements summing to 1; in symbols, we have

$$X_{tj}|Y_{t-1}=y_{t-1},\beta_{j}∼\text{Mult}(y_{t-1,j};p_{tj}),$$

for $t\in {𝒯}^{\prime }$ and j∈𝒦, where $\beta_{j}$ is the matrix of the regression vectors $\beta_{jk}$, $k\in {𝒟}_{j}$, that are involved in the model for the probabilities in $p_{tj}$ as will be clarified below; see Equation (3). In particular, $p_{tjk}$ is the conditional probability that an individual is in category *k* at occasion *t* given that he/she was in category *j* at the previous time occasion. Assuming the multinomial distribution we can write

(1) $$p(X_{tj}=x_{tj}|Y_{t-1}=y_{t-1},\beta_{j})=\overset{K}{\underset{k=1}{\prod }}\frac{y_{t-1,j}!}{x_{tjk}!}p_{tjk}^{x_{tjk}}.$$

It is well known that this conditional probability is related to the Poisson distribution. In particular, it is the conditional distribution of a set of independent random variables having Poisson distribution given their total.^29^ The conditional expected value and the variance‐covariance matrix under the multinomial distribution have the following expressions:

$$\begin{array}{ll} E(X_{tj}|Y_{t-1}=y_{t-1},\beta_{j}) & =y_{t-1,j}p_{tj},\;\\ \mathrm{Var}(X_{tj}|Y_{t-1}=y_{t-1},\beta_{j}) & =y_{t-1,j}[\text{diag}(p_{tj})-p_{tj}p_{tj}^{\prime }].\; \end{array}$$

In order to account for overdispersion, which may arise in the count data, we also consider a Dirichlet‐multinomial distribution^29^, ^30^, ^31^ for each vector $X_{tj}$ given $Y_{t-1}$, which is denoted by

$$X_{tj}|Y_{t-1}=y_{t-1},\beta_{j}∼\mathrm{Dir}-\mathrm{Mult}(y_{t-1,j};\alpha_{tj}),$$

for $t\in {𝒯}^{\prime }$ and j∈𝒦, and depends on a vector of parameters $\alpha_{tj}={\left(\alpha_{tj1},\ldots ,\alpha_{tjK}\right)}^{\prime }$. Consequently, we have that

(2) $$p(X_{tj}=x_{tj}|Y_{t-1}=y_{t-1},\beta_{j})=\frac{y_{t-1,j}!\Gamma (\alpha_{tj+})}{\Gamma (y_{t-1,j}+\alpha_{tj+})}\overset{K}{\underset{k=1}{\prod }}\frac{\Gamma (x_{tjk}+\alpha_{tjk})}{x_{tjk}!\Gamma (\alpha_{tjk})},$$

where $\alpha_{tj+}=\sum_{k\in 𝒦}\alpha_{tjk}$, so that

$$\begin{array}{lcr} E(X_{tj}|Y_{t-1}=y_{t-1},\beta_{j}) & = & y_{t-1,\;j}\frac{\alpha_{tj}}{\alpha_{tj+}},\\ \mathrm{Var}(X_{tj}|Y_{t-1}=y_{t-1},\beta_{j}) & = & y_{t-1,\;j}\left[\text{diag}\left(\frac{\alpha_{tj}}{\alpha_{tj+}}\right)-\frac{\alpha_{tj}}{\alpha_{tj+}}\frac{\alpha_{tj}^{\prime }}{\alpha_{tj+}}\right]\frac{y_{t-1,\;j}+\alpha_{tj+}}{1+\alpha_{tj+}}. \end{array}$$

Parameters collected in $\beta_{j}$ affect the parameters $\alpha_{tjk}$ as will be clarified below. Note that the level of overdispersion decreases as the total $\alpha_{tj+}$ increases. This overdispersion may be motivated by the presence of measurement errors or unobserved heterogeneity, as the national counts are obtained by collapsing counts referred to different regions. Moreover, it is possible that, within our formulation, we omit important covariates because they are not available to us. These missing covariates may act as risk factors and influence the observed counts.

Obviously, either if we assume a multinomial or a Dirichlet‐multinomial distribution, formulated in (1) or (2), respectively, the induced distribution for $Y_{t}$ given $Y_{t-1}$ has a complex expression involving the sum of quantities like

$$\underset{j\in 𝒦}{\prod }p(X_{tj}=x_{tj}|Y_{t-1}=y_{t-1},\beta_{j})$$

over all possible configurations of the contingency table with frequencies $x_{t1},\ldots ,x_{tK}$ having certain column totals. In the multinomial case, the induced distribution is related to the multivariate hypergeometric that, however, may be difficult to compute in practice.

### 2.2 Adopted parametrizations

Under the multinomial model, in order to parametrize each probability vector $p_{tj}$ we introduce the subsets ${𝒟}_{j}$ of 𝒦 containing the indices of the elements of this vector that are not constrained to be equal to zero. Then, we assume the multinomial logit parametrization

(3) $$p_{tjk}=\frac{\exp(f_{tjk}^{\;\prime }\beta_{jk})}{\sum_{l\in {𝒟}_{j}}\exp(f_{tjl}^{\;\prime }\beta_{jl})},\;t\in {𝒯}^{\prime },j\in 𝒦,k\in {𝒟}_{j},$$

where the design column vectors $f_{tjk}$ contain the terms of a suitable polynomial of time *t* included in the model via the regression parameter vector $\beta_{jk}$. These parameters may be interpreted in terms of the logit of the probability of moving to category *k* starting from category *j*.

To ensure model identifiability, under the multinomial distribution we assume that $\beta_{jj}\equiv 0$, j∈𝒦, where 0 is a suitable dimensional column vector of zeros. In our application, in particular, we use common vectors across categories containing the elements of a second or third order polynomial, and we have $f_{tjk}={\left(1,t,t^{2},t^{3}\right)}^{\prime }$ for all *t*, *j*, and *k*. These vectors may also include covariates, such as dummies, to study the effect of epidemic containment policies^16^ imposed at a certain time for mitigating the pandemic, as we show in our application. Alternatively, proper splines^32^, ^33^ may be considered. Overall, the free parameters of the multinomial model are collected in the matrix β; in particular, this matrix collects the vectors $\beta_{jk}$, j∈𝒦, $k\in {𝒟}_{j}^{\prime }$, where ${𝒟}_{j}^{\prime }={𝒟}_{j}\setminus \{j\}$.

The parametrization of the Dirichlet‐multinomial version of the proposed model is simpler as we directly assume that

(4) $$\alpha_{tjk}=\exp(f_{tjk}^{\;\prime }\beta_{jk}),\;t\in {𝒯}^{\prime },j\in 𝒦,k\in {𝒟}_{j},$$

on the basis of the quantities already defined above. In this case we not need to introduce identifiability constraints on the $\beta_{jk}$ parameters and then we define the overall parameter matrix β as that collecting the vectors $\beta_{jk}$, j∈𝒦, $k\in {𝒟}_{j}$. The corresponding probabilities may be computed as

(5) $$p_{itk}=\frac{\exp(\alpha_{tjk})}{\sum_{l\in {𝒟}_{j}}\exp(\alpha_{tjl})},\;t\in {𝒯}^{\prime },j\in 𝒦,k\in {𝒟}_{j}.$$

In a Bayesian framework, we assume that a priori the regression parameters in each vector $\beta_{jk}$ are independent and have a diffuse prior distribution. The initial and most natural choice is that of a multivariate Gaussian distribution, that is,

(6) $$\beta_{jk}∼N(0,\sigma^{2}I),\;j\in 𝒦,$$

where $k\in {𝒟}_{j}^{\prime }$ for the multinomial version and $k\in {𝒟}_{j}$ for the Dirichlet‐multinomial case, I is a suitable dimensional identity matrix, and $\sigma^{2}$ is the variance hyperparameter that can be fixed to be large, in case we want to assume a noninformative prior to perform exploratory analysis; for instance, we use a value equal to 100 in our application. However, in order to include certain epidemiological hypotheses in the model, such as the fact that some transitions are very rare or even impossible, and in order to increase the numerical stability of estimation and prediction, we introduce inequality constraints on convenient transformations of the parameters. These constraints can be used in a very flexible way and may be reduced to equality constraints. Let $o_{tjk}$ be the odds referred to category *k* with respect to category *j* at time occasion *t*, which are defined as $o_{tjk}=p_{tjk}/p_{tjj}$. Our approach allows us to include the constraint that, for selected pairs of indices (j,k) in the set 𝒞 to be appropriately chosen, for all time occasions *t* these odds are bounded from above and/or below although, in the application illustrated in Section 4, we only use upper limits. More precisely, we assume that

(7) $$a_{jk}\leq o_{tjk}\leq b_{jk},\;t\in {𝒯}^{*},(j,k)\in 𝒞,a_{jk},b_{jk}\in R^{+},$$

where ${𝒯}^{*}=\{2,\ldots ,T^{*}\}$ and $T^{*}$ refers to the time until which predictions are computed. In summary, from a Bayesian perspective, we can also assume truncated priors^34^ based on the multivariate Gaussian distributions in (6) under the constraint given in (7). Then, the model prior formulation amounts to specify the value of the variance $\sigma^{2}$ together with the set 𝒞 and the limits $a_{jk}$ and $b_{jk}$ for the odds having indices in this set.

An alternative to the approach to formulate prior distributions described above could rely on a reparametrization ensuring that inequalities in (7) are always attained, so that a prior Gaussian distributions can be assumed on the transformed parameters without introducing any truncation. However, we prefer to retain the proposed way to specify prior distributions as the above inequalities can be easily accounted for in the MCMC estimation algorithm.

Possible extensions of the above formulation could consist in differentiating the order of the polynomial for the multinomial or Dirichlet‐multinomial parameters considered in (3) and (4) across the possible pairs (j,k), but we prefer the approach based on truncated priors to avoid model selections issues and because the proposed truncation is based on a clearly interpretable criterion. Moreover, in the proposed framework, it is also possible to use informative prior distributions for the parameter vectors $\beta_{jk}$. In particular, for each of these vectors we can assume a Gaussian prior distribution with mean vector and variance‐covariance matrix equal to the posterior estimates obtained from data available in countries that entered earlier in the pandemic emergency phase. In this way, we can have more accurate predictions at the beginning of the pandemic, when only data referred to a few time occasions are available.

### 2.3 Comparison with alternative models

The main employed models to predict the expected number of infections use univariate counts assumed to follow a Poisson or a negative binomial distribution. However we jointly model all the observable counts while the full process of transitions between categories is unobservable. An approach of this type for 2×2 contingency tables has been proposed in Reference 11 on the basis of a Binomial distribution assumed for each row of these tables. Even this approach follows a Bayesian formulation based on Beta prior distributions assumed for suitable probability parameters and it relies on an MCMC estimation algorithm, with special attention to inference on the odds ratio as a measure of association in each contingency table.

It is worth noting that our proposal may be cast in the literature on hidden Markov (HM) model.^35^, ^36^, ^37^ A model of this class was first introduced to monitor epidemiological surveillance data for poliomyelitis counts.^38^ However the literature in this field is not rich and this model is generally estimated by considering a penalized likelihood approach where the choice of the penalty is crucial. In our context, we consider a simpler model avoiding the definition of the latent states and we propose a fully Bayesian formulation by considering an MCMC algorithm which allows us to dispose of the simulated posterior probabilities of the model parameters.

Similarly to the Poisson model, our model has an epidemiological interpretation in line with the more common SEIR models^12^ and we also provide an estimate of the reproduction number $R_{t}$ defined as the expected number of individuals a single infected person will infect over the course of his/her infection period. The $R_{t}$ can be considered as the average number of secondary cases per primary case; see Reference 39 for a study on the differences between the estimation of $R_{t}$ in a deterministic SEIR‐type model and in a stochastic model like the Poisson model. A first attempt to estimate this number for COVID‐19 is provided by Shao et al^40^ and for Italy by Cereda et al^17^ among others.

## 3 BAYESIAN INFERENCE

In this section we provide some details about estimation of the parameters and of the reproduction number. We also deal with methods for model checking and for model comparison across different specifications.

### 3.1 Parameter estimation

The model is estimated through a Metropolis sampler by implementing a fixed scan algorithm based on two steps that are iteratively repeated. In the first step, we update the contingency tables $X_{t}$ with elements $X_{tjk}$, j,k∈𝒦, given the current value of the parameters and the observed margins $y_{t-1}$ and $y_{t}$, for $t\in {𝒯}^{\prime }$. In the second step we update the model parameters $\beta_{jk}$ and an MH ratio is computed for each parameter vector in order to decide if the candidate move may be accepted.

Given the complexity of sampling tables with fixed margins under the assumption that frequencies in each row of the contingency table follow a multinomial or a Dirichlet‐multinomial distribution with parameters defined in (3) or (5), we use the technique of Reference 41 based on: (*i*) randomly selecting two rows and two columns of the current table so that a 2×2 subtable is identified; (*ii*) performing a switch that consists in adding (or subtracting) to the two cells in the main diagonal of the subtable a random integer number that is subtracted (or added) to the off‐diagonal cells; (*iii*) provided that the table proposed on the basis of the random switch has all positive frequencies, accepting this table with probability equal to

$$\alpha =\min\left(1,\underset{j\in 𝒦}{\prod }\frac{p(X_{tj}=x_{tj}^{*}|Y_{t-1}=y_{t-1},\beta_{j})}{p(X_{tj}=x_{tj}|Y_{t-1}=y_{t-1},\beta_{j})}\right),$$

where $x_{tj}$ is the vector of the frequencies in the *j*th row of the current table, $x_{tj}^{*}$ is that of the proposed table, and $\beta_{j}$ is the matrix containing all current regression vectors $\beta_{jk}$, $k\in {𝒟}_{j}$. On the basis of the definition of the probabilities involved in the expression given in (1) or (2), several simplifications are possible in computing the acceptance MH ratio.

After having updated the tables, and when the multinomial formulation is adopted, for each j∈𝒦 and $k\in {𝒟}_{j}^{\prime }$, we update the regression parameters with a random walk Metropolis step and propose a new value of $\beta_{jk}$ from a normal distribution centered on the current value of this parameter vector and with a proper variance‐covariance matrix. Then, provided that inequalities in are verified (7), the proposed vector, denoted by $\beta_{jk}^{*}$ is accepted with probability

$$\alpha =\min\left(1,\frac{\prod_{t\in {𝒯}^{\prime }}p(X_{tj}=x_{tj}|Y_{t-1}=y_{t-1},\beta_{jk}^{†})}{\prod_{t\in {𝒯}^{\prime }}p(X_{tj}=x_{tj}|Y_{t-1}=y_{t-1},\beta_{j})}\frac{\pi (\beta_{jk}^{*})}{\pi (\beta_{jk})}\right),$$

where $\beta_{jk}^{†}$ is the same matrix as $\beta_{j}$ with $\beta_{jk}$ substituted with $\beta_{jk}^{*}$, and $\pi (\beta_{jk})$ is the prior density of the regression parameters. For the Dirichlet‐multinomial version, the updating step of the $\beta_{jk}$ parameters is performed as above for each $k\in {𝒟}_{j}$.

At the end of the algorithm we obtain the simulated tables $X_{t}^{\;(s)}$, $t\in {𝒯}^{\prime }$, and the parameter vectors $\beta_{jk}^{\left(s\right)}$ drawn from the posterior distribution, for s=1,…,S, where *S* is the number of MCMC iterations. At every iteration we also include estimation and prediction of the reproduction number $R_{t}$, as we detail in Section 3.3.

### 3.2 Frequency prediction

After having updated tables and regression parameters, at each iteration of the MCMC algorithm, we also make in‐sample and out‐sample prediction of the frequencies $y_{tk}$. In particular, the MCMC algorithm draws parameter vectors $\beta_{jk}^{\left(s\right)}$ on the basis of which it is possible to obtain the probabilities $p_{tjk}^{\left(s\right)}$ computed according to Equation (3) or (5) depending on the assumed count distribution, multinomial or Dirichlet‐multinomial. Consequently, a prediction of the frequency $y_{tk}$ at step *s* of the algorithm is given by

$${ŷ}_{tk}^{\left(s\right)}=\underset{j\in 𝒦}{\sum }\;y_{t-1,j}p_{tjk}^{\left(s\right)},\;t\in 𝒯.$$

The same formula may be applied for predicting $y_{tk}$ for t=T+1, obtaining ${ŷ}_{T+1,k}$, whereas we can apply the recursive formula

$${ŷ}_{tk}^{\left(s\right)}=\underset{j\in 𝒦}{\sum }\;{ŷ}_{t-1,j}^{\left(s\right)}p_{tjk}^{\left(s\right)}$$

for t>T+1. These predictions are collected in vectors ${\hat{y}}_{t}^{\left(s\right)}$ in a suitable way.

An alternative way to perform out‐sample predictions of the frequencies $y_{tk}$ is based on simulating, at each step, the table for occasion T+1, denoted by $X_{T+1}^{\;(s)}$ for iteration *s*, from the assumed distribution on the basis of the current parameter values and the observed frequencies in the vector $y_{T}$. By summing the columns of table $X_{T+1}^{\;(s)}$ we obtain the predicted frequencies ${\tilde{y}}_{T+1,k}^{\left(s\right)}$ collected in vector ${\tilde{y}}_{T+1}^{\left(s\right)}$. This process is performed recursively so as to obtain the simulated tables $X_{t}^{\;(s)}$ on the basis of the frequencies ${\tilde{y}}_{t-1}^{\left(s\right)}$ and the corresponding predicted frequencies ${\tilde{y}}_{tk}^{\left(s\right)}$ collected in vectors ${\tilde{y}}_{t}^{\left(s\right)}$ for t>T+1.

At the end of the algorithm we can obtain summary statistics for the predictions ${ŷ}_{tk}^{\left(s\right)}$ and ${\tilde{y}}_{tk}^{\left(s\right)}$ starting from simple means across the iterations, denoted by ${ŷ}_{tk}$ and ${\tilde{y}}_{tk}$, respectively. We can also associate measures of precision that take into account the variance of the posterior parameter distribution. In particular, by computing the variance of the ${\tilde{y}}_{tk}^{\left(s\right)}$ predictions, we appropriately measure the level of uncertainty of such predictions that are directly generated from the model.

### 3.3 Estimation of a time‐evolving reproduction number

In order to estimate the net reproduction number $R_{t}$ we take inspiration from the method already applied to the Italian context^17^, ^42^ and that is rather popular in epidemiology; see Reference 43, among others. In particular, we start from the assumption that the “serial interval” for COVID‐19 follows a Gamma distribution with parameters 1.87 and 0.28, so that the mean is of 6.6 days, as established in Reference 17. However, note that from the literature uncertainty emerges about the length of the serial interval, as highlighted in the meta‐analysis provided in Reference 44. The assumed length of this interval may strongly affect the final estimate of $R_{t}$. Still, the reference model we select for the serial interval has been already used as a standard value for Italian data, and we thus adopt it for uniformity and comparability purposes.

At every iteration *s* of the MCMC algorithm described in Section 3.1 we predict the reproduction number at issue as

$${\hat{R}}_{t}^{\left(s\right)}=\frac{{\hat{\Delta I}}_{t}^{\left(s\right)}}{\sum_{r=1}^{t-1}\omega_{s,t-1}{\hat{\Delta I}}_{t-r}^{\left(s\right)}},$$

where $\omega_{r,t-1}$ is a weight obtained by normalizing the density of the Gamma distribution with the above parameters (1.87 and 0.28) so that $\sum_{r=1}^{t-1}\omega_{r,t-1}=1$ and ${\hat{\Delta I}}_{t}^{\left(s\right)}$ is the number of individuals in category NP predicted by the model for day *t*. The latter is directly computed on the basis of the sum of suitable elements of the transition probability matrix from the first category, namely,

$${\hat{\Delta I}}_{t}^{\left(s\right)}=y_{t-1,1}\overset{K}{\underset{k=3}{\sum }}p_{t1k}^{\left(s\right)}.$$

Finally, we take the overall prediction as a mean across the MCMC iterations. These means are denoted by ${\hat{R}}_{t}$. This procedure allows us to estimate the net reproduction number for the observed time occasions and out of sample. We may also obtain a measure of precision and credible intervals to be associated with the predicted $R_{t}$ values, also accounting for the variability of the parameter estimators.

Overall, the method we adopt to estimate the reproductive number presents elements of novelty with respect to the previous proposals that require the use of an ad hoc MCMC algorithm involving a likelihood function for the counts of NP individuals based on the Poisson distribution.

### 3.4 Model checking and comparison

From the MCMC algorithm we obtain the predicted frequencies ${ŷ}_{tk}^{\left(s\right)}$ and ${\tilde{y}}_{tk}^{\left(s\right)}$ as illustrated at the end of Section 3.2. In order to evaluate the goodness‐of‐fit of the model we then consider the following discrepancy measure

(8) $${\hat{\text{Dist}}}^{\left(s\right)}=\underset{t\in {𝒯}^{\prime }}{\sum }\underset{k\in 𝒦}{\sum }\frac{{\left(y_{tk}-{ŷ}_{tk}^{\left(s\right)}\right)}^{2}}{{ŷ}_{tk}^{\left(s\right)}},$$

which is computed at every MCMC iteration *s*. The overall measure of fit of the model may then be obtained as the mean of these quantities across the MCMC iterations, obtaining $\hat{\text{Dist}}$. In order to calculate the corresponding posterior predictive *p*‐value we follow the procedure illustrated in Reference 26, see also References 27 and 45. In particular, for every iteration, we also compute a version of the discrepancy measure, denoted by ${\tilde{\text{Dist}}}^{\left(s\right)}$, using formula (8) but with each observed frequency $y_{tk}$ substituted by a simulated frequency from the model with the current parameter value and based on the previously observed frequencies $y_{t-1,j}$. The mean of these statistics across iterations is denoted by $\tilde{\text{Dist}}$. Then the posterior predictive *p*‐value is obtained as the proportion of times that ${\tilde{\text{Dist}}}^{\left(s\right)}$ is at least equal to ${\hat{\text{Dist}}}^{\left(s\right)}$ across all the MCMC iterations. Although this procedure has been criticized because the observed data are used twice, once for parameter estimation and once for model checking, the resulting posterior predictive *p*‐values is still a useful check of model fit provided that it is correctly interpreted. In particular if the model has an adequate fit, then *p*‐values close to 0.5 should be observed.^45^

The above discrepancy measure may also be used to compare different models when they are used for obtaining forecasts and for this aim, we consider an out‐sample version that is time specific. In particular suppose that further observations are available with respect to those used to estimate the model, that is, suppose we know $y_{tk}$ for $t\in {𝒯}^{†}$, where ${𝒯}^{†}=\{T+1,\ldots ,T^{†}\}$ with $T^{†}>T$. Then, at every MCMC iteration we compute the discrepancy measure

(9) $${\hat{\text{Dist}}}_{t}^{\left(s\right)}=\underset{k\in 𝒦}{\sum }\frac{{\left(y_{tk}-{ŷ}_{tk}^{\left(s\right)}\right)}^{2}}{{ŷ}_{tk}^{\left(s\right)}},\;t\in {𝒯}^{†},$$

which is again summarized by a mean denoted by ${\hat{\text{Dist}}}_{t}$ and for which we obtain an out‐of‐sample posterior *p*‐value according to the same method illustrated above. In this case, using different data for parameter estimation and model checking we consider *p*‐values larger than 0.05 as adequate.

Finally, it may also be of interest to understand with respect to which categories, among the *K* considered, the proposed approach presents a higher or lower performance in terms of forecasting. In this regard we use the following type of discrepancy measure

(10) $${\tilde{\text{Dist}}}_{k}^{*}=\underset{t\in {𝒯}^{†}}{\sum }\frac{{\left(y_{tk}-{\tilde{y}}_{tk}\right)}^{2}}{{\tilde{y}}_{tk}},\;k\in 𝒦.$$

Note that in this case we directly use the predictions available at the end of the algorithm, and we compare them with those produced by sampling from the assumed distribution and denoted as ${\tilde{y}}_{tk}$.

## 4 APPLICATION

Following the spread of COVID‐19 in Europe, we consider the daily counts for K=6 categories illustrated at the beginning of Section 2 and denoted by S, R, Q, H, ICU, and D. Results are shown with reference to the Italian data collected from 24 February until 24 April 2020 (day 61) in order to evaluate the performance of our proposal at the beginning of the pandemic. First, we compare the goodness‐of‐fit of the estimated models, and then we report the results of the best model along with some results of another model for comparison. Then in Section 4.2 we show additional results obtained with data collected on the same period referred to individuals who reside in the Lombardy region where the Italian wave of the epidemic started. In the Appendix we provide some additional details on the estimation algorithm, data, and codes.

### 4.1 Italian data

#### 4.1.1 Model comparison

We started our analysis with a variety of models formulated according to the proposed approach. In particular, we considered both the multinomial and the Dirichlet‐multinomial autoregressive versions with polynomials of order two or three and with or without constraints on the odds, as formulated in (7). The constraints imposed on the odds for the transitions between categories ($o_{tjk}$) are displayed in Table 2, reporting the maximum values that these odds can take ($b_{jk}$). For example, in Table 2 the odds for the transition from ICU to H is 0.25, meaning that the probability to move from ICU to H can be at most one fourth of that of remaining in ICU in a given day.

**TABLE 2:** Table of the fixed upper bounds for the odds of the transitions between categories

	S	R	Q	H	ICU	D
**S**	—	10${}^{-7}$	0.001	10${}^{-4}$	10${}^{-6}$	10${}^{-7}$
**R**	—	—	0.001	10${}^{-4}$	10${}^{-6}$	10${}^{-7}$
**Q**	—	0.1	—	0.1	10${}^{-5}$	10${}^{-6}$
**H**	—	0.1	0.1	—	0.1	0.01
**ICU**	—	10${}^{-7}$	10${}^{-7}$	0.25	—	0.25
**D**	—	—	—	—	—	—

Overall, we considered eight models by combining two distributions (multinomial or Dirichlet‐multinomial), two orders of the polynomial (two or three), and two specifications (with or without constraints). All these models included two dummy variables to account for the effect of NPI enforced on 24 February and on 8 March 8 2020, aimed at containing viral transmission by closing schools, limiting movements, and imposing social distancing. Two dummy variables have been added on days 7 and 20, corresponding to the 1st and 14th of March, considering that the effects of these NPIs can be detected approximately 1 week after their enforcement.

In order to compare the eight models we used the discrepancy measures illustrated in Section 3.4. In Table 3 we report the observed values of statistics $\hat{\text{Dist}}$ and $\tilde{\text{Dist}}$ and the corresponding average posterior predictive *p*‐values. These results suggest that the multinomial model shows a lack of fit, a problem that is resolved in the model based on the Dirichlet‐multinomial distribution. In particular, all the Dirichlet‐multinomial autoregressive models have a much better fit with respect to the models based on the multinomial distribution: they are more capable of reproducing the amount of variation observed in the data. Among the models based on the Dirichlet‐multinomial distribution, Model 7 is the best to explore the information contained in the data and has a posterior predictive *p*‐value very close to 0.5 as expected when the model is adequate. In contrast Model 8, imposing upper limits on the odds as those illustrated in Table 2, is suitable to provide an epidemiological interpretation in line with the most common SEIR epidemiological models.^12^

**TABLE 3:** Average realized and predicted discrepancy measures for the autoregressive multinomial and Dirichlet‐multinomial models and posterior predictive *p*‐values for Italian data

Autoregressive model			
Multinomial	$\hat{\text{Dist}}$	$\tilde{\text{Dist}}$	*p*‐value
Model 1 (2nd order, without constraints)	1658.011	124.670	0.000
Model 2 (2nd order, with constraints)	2347.274	68.474	0.000
Model 3 (3rd order, without constraints)	1565.587	122.793	0.000
Model 4 (3rd order, with constraints)	2203.832	70.512	0.000
**Dirichlet‐multinomial**	$\hat{\text{Dist}}$	$\tilde{\text{Dist}}$	***p*‐value**
Model 5 (2nd order, without constraints)	2608.502	3060.236	0.679
Model 6 (2nd order, with constraints)	2992.213	3629.419	0.750
Model 7 (3rd order, without constraints)	2414.970	2811.524	0.536
Model 8 (3rd order, with constraints)	2915.772	3344.208	0.661

We forecasted the total number of reported cases according to the posterior predictive distribution over the course of 10 days after the estimation time window and compared them with the observed cases during these days. Table 4 shows the realized values of the proposed discrepancy measure defined in (9) and ${\tilde{\text{Dist}}}_{t}$, which is based on the simulated frequency along with the corresponding posterior *p*‐value for each predicted day resulting from Models 7 and 8. We observe that for Model 8 the *p*‐values are never less than 0.05. Moreover, as expected, the overall *p*‐value decreases with the increasing number of predicted days.

**TABLE 4:** Realized values of the discrepancy measures according to Models 7 and 8 for the forecasted cases in Italy and posterior *p*‐values over a period of 10 days

	Model 7			Model 8		
Day	${\hat{\text{Dist}}}_{t}$	${\tilde{\text{Dist}}}_{t}$	*p*‐value	${\hat{\text{Dist}}}_{t}$	${\tilde{\text{Dist}}}_{t}$	*p*‐value
25th April	18.971	9.128	0.202	7.383	23.344	0.769
26th April	200.800	16.790	0.003	60.573	44.372	0.403
27th April	596.703	23.242	0.000	2200.393	63.880	0.198
28th April	1202.657	28.746	0.000	335.829	81.942	0.161
29th April	2222.529	33.588	0.000	505.395	98.120	0.137
30th April	2664.510	37.776	0.000	434.868	113.028	0.164
1st May	4779.427	41.501	0.000	658.215	127.831	0.118
2nd May	8358.893	44.679	0.000	929.957	140.980	0.103
3rd May	13544.235	47.837	0.000	1219.478	153.172	0.095
4th May	21402.362	51.219	0.000	1767.593	165.840	0.069

In Table 5 we also report the measure provided in (10) for both models and notice that the best predicted categories are D and ICU. Category H is also very well predicted. This is an important feature of the model since ICU is the crucial count to correctly predict in order to save lives by optimal management of health‐care resources. In the following, we provide more details on the results obtained with the selected models, starting from Model 8 that incorporates constraints and provides a more straightforward epidemiological interpretation.

**TABLE 5:** Realized values of the discrepancy measure for each category referred to the observed and predicted counts over a period of 10 days

	S	R	Q	H	ICU	D	Total
Model 7 ${\tilde{\text{Dist}}}_{k}^{*}$	8.000	28 507	12 926	3527	177	339	45 484
Model 8 ${\tilde{\text{Dist}}}_{k}^{*}$	0.000	1409	1397	372	31	12	3220

#### 4.1.2 Results of obtained from Model 8

**FIGURE 1:**
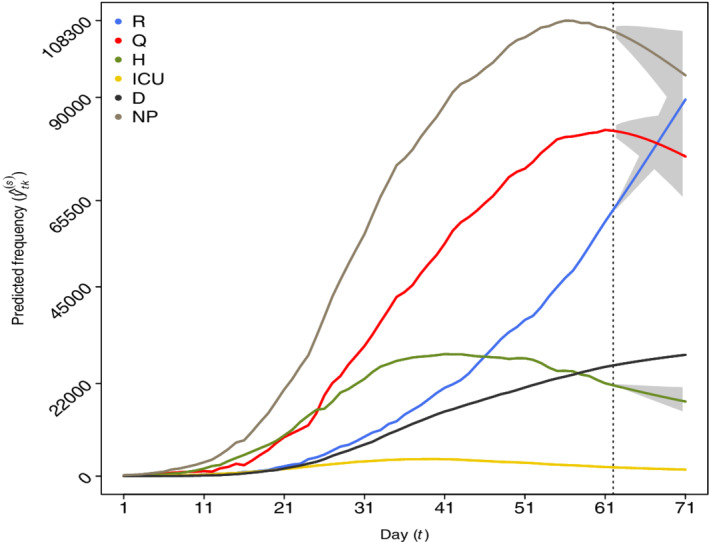
Observed frequencies (before the vertical dashed line corresponding to the 25th of April) and 10 days predictions (after the vertical line until the 4th of May) with Model 8 for categories: recovered (R), positive cases in quarantine (Q), hospitalized (H), intensive care (ICU), deceased (D), and “now positive” (NP). The estimated 95% prediction intervals are visualized in gray [Colour figure can be viewed at wileyonlinelibrary.com]

Figure 1 shows the daily observed and predicted counts for each category with a time horizon of 10 days and the estimated 95% prediction intervals depicted in gray.

The posterior means of the predicted transition frequencies referred to the 25th and the 26th of April, stored in the transition matrix, are reported in Table 6. The corresponding 95% upper and lower predicted limits are reported in Table 7. The configuration of the predicted frequencies in Table 6 is recovered from the fixed marginal frequency of total counts. We observe that some transitions are absent and that the highest frequency is predicted for the transition from S to Q (2219 individuals), and the second highest transition is predicted from H to Q (757 individuals). Some patients are predicted to transit from Q to H (516) and from H to ICU (73).

For 26 April compared with 25 April, it is estimated that 149 deaths are expected among ICU patients, with a credibility interval from 123 to 282. These estimates imply an average length of stay in ICU ranging from 10 to 22 days. Another interesting observation is that on the same day of 26 April, 73 hospitalized patients (0.33%) are predicted to require intensive care with a credibility interval of 25 to 137 patients (0.11%, 0.62%). On the other hand, 197 hospitalized patients are predicted to die on the same day.

**TABLE 6:** Estimated posterior means of the predicted transitions between categories obtained with Model 8 from 25th to 26th of April (from the 61st to the 62nd day)

	S	R	Q	H	ICU	D
**S**	60 121 632	0	2219	154	1	0
**R**	0	60 489	9	0	0	0
**Q**	0	2665	79 105	516	0	0
**H**	0	116	757	20 925	73	197
**ICU**	0	0	0	0	2023	149
**D**	0	0	0	0	0	25 969

**TABLE 7:** Estimated posterior 95% prediction upper and lower bounds for the transitions between categories obtained with Model 8 from 25th to 26th of April (from the 61st to the 62nd day)

	S	R	Q	H	ICU	D
**S**	—	(0, 0)	(1217, 3188)	(0, 718)	(0, 2)	(0, 0)
**R**	—	(60 471, 60 498)	(0, 26)	(0, 0)	(0, 0)	(0, 0)
**Q**	—	(1269, 4357)	(77 182, 80 672)	(32, 1479)	(0, 0)	(0, 0)
**H**	—	(0, 506)	(463, 1129)	(20 438, 21 321)	(25, 137)	(123, 282)
**ICU**	—	(0, 0)	(0, 0)	(0, 40)	(1963, 2075)	(98, 210)
**D**	—	—	—	—	—	—

The estimated posterior means and the 95% predicted interval for the increase in totals for H and ICU from 26th to 29th April are reported in Table 8. These are of particular interest, since during the first period of the pandemic, there was a significant daily increase in the demand for hospital beds and ICU, especially from the general population at risk of being affected by the virus.

**TABLE 8:** Estimated posterior means and 95% prediction intervals (PI) of the increase in totals for H and ICU over a period of 10 days obtained with Model 8

Day	H	PI	ICU	PI
25th April	−472	(−1047, 446)	−76	(−140, −2)
26th April	−465	(−1032, 462)	−73	(−134, −2)
27th April	−460	(−1012, 459)	−69	(−128, −1)
28th April	−450	(−997, 465)	−67	(−122, 0)
29th April	−442	(−981, 470)	−63	(−118, 0)
30th April	−431	(−972, 450)	−60	(−112, 2)
1st May	−420	(−952, 465)	−57	(−107, 3)
2nd May	−409	(−948, 459)	−55	(−104, 4)
3rd May	−397	(−942, 484)	−52	(−99, 6)
4th May	−384	(−925, 454)	−50	(−95, 7)

**FIGURE 2:**
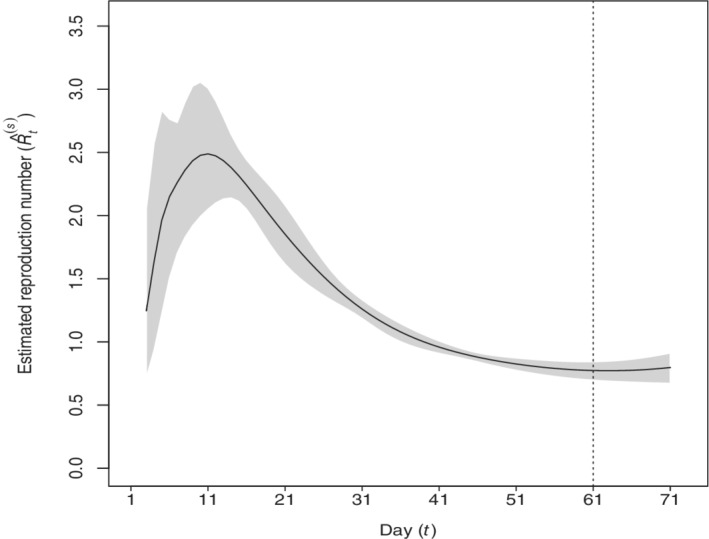
Estimated and predicted (from the vertical line) reproduction number $R_{t}$ obtained with Model 8 (61 observed days, prediction from 25th of April to 4th of May). The estimated 95% credibility and prediction intervals are displayed in gray

In order to show the temporal dynamics of the estimated daily reproduction number $R_{t}$, Figure 2 depicts the estimated averages and 95% credible bounds obtained using all the data of the 61 days along with the predicted values for 10 days. From this figure we observe that, on average, this value increases over time during the early phase of the epidemic before containment measures became effective, and it only begins to decrease on the 11th day, corresponding to the 5th of March. Trend and values resemble those provided by the Italian National Institute of Health.^42^

#### 4.1.3 Some results obtained from Model 7

In the following, we show some results obtained from Model 7 for Italy since, as stated above, this model is reasonable, especially for exploratory data purposes, as no constraints are imposed on the odds for the transitions across categories. The posterior means of the predicted transition frequencies referred to 25th and 26th April stored in the transition matrix are reported in Table 9. Comparing this table with Table 6 we note some differences, for example, the fact that 1243 people are predicted to transit from S to R; however, this transition is rather implausible. This confirms that, as stated above, the proposed constraints are suitable to comply with the epidemiological features of the pandemic.

**TABLE 9:** Estimated posterior means of the predicted transitions between categories 25th to 26th of April (from the 61st to the 62nd day) obtained with Model 7

	S	R	Q	H	ICU	D
**S**	60 121 106	1243	1632	22	4	0
**R**	0	58 106	2278	0	42	71
**Q**	0	3 461	76 798	1675	22	330
**H**	0	1155	1228	19 617	2	66
**ICU**	0	1	139	2	2029	1
**D**	0	0	0	0	0	25 969

The estimated posterior mean and the 95% predicted interval for the increase in totals for H and ICU from the 26th to the 29th April are reported in Table 10. Comparing this table with Table 8 we notice that the main difference is observed for the predicted frequencies for category H, and, as expected, these intervals are wider.

**TABLE 10:** Estimated posterior means and 95% prediction intervals (PI) for 10 days of the increase in totals for H and ICU obtained with Model 7

Day	H	PI	ICU	PI
25th April	−752	(−1486, −64)	−75	(−132, 10)
26th April	−800	(−1570, −80)	−69	(−130, 20)
27th April	−847	(−1626, −116)	−62	(−127, 37)
28th April	−898	(−1711, −145)	−54	(−125, 55)
29th April	−948	(−1810, −160)	−46	(−126, 80)
30th April	−996	(−1901, −191)	−37	(−128, 111)
1st May	−1042	(−1959, −205)	−27	(−134, 150)
2nd May	−1083	(−2021, −229)	−16	(−141, 199)
3rd May	−1116	(−2041, −241)	−3	(−151, 258)
4th May	−1140	(−2041, −246)	13	(−155, 349)

### 4.2 Lombardy data

We show additional results obtained when the proposed models are estimated with data referred to the Lombardy region.

#### 4.2.1 Model comparison

The realized values of the discrepancy measures of the eight models estimated with the available data are reported in Table 11. We note that, as for the Italian data, the Dirichlet‐multinomial autoregressive models are more suitable to explain the variability observed in the data with respect to the models based on the multinomial distribution. The posterior predictive *p*‐value closer to 0.5 is the one calculated for Model 8.

**TABLE 11:** Average realized and predicted discrepancy measures for the autoregressive multinomial and Dirichlet‐multinomial models, average posterior two‐sided *p*‐values for data from Lombardy

Autoregressive model			
Multinomial	$\hat{\text{Dist}}$	$\tilde{\text{Dist}}$	*p*‐value
Model 1 (2nd order, without constraints)	2487.417	147.896	0.000
Model 2 (2nd order, with constraints)	3868.846	70.011	0.000
Model 3 (3rd order, without constraints)	2532.507	139.870	0.000
Model 4 (3rd order, with constraints)	3855.632	71.237	0.000
**Dirichlet‐multinomial**	$\hat{\text{Dist}}$	$\tilde{\text{Dist}}$	***p*‐value**
Model 5 (2nd order, without constraints)	4487.878	5911.391	0.765
Model 6 (2nd order, with constraints)	5210.567	4960.395	0.388
Model 7 (3rd order, without constraints)	4346.473	6514.379	0.646
Model 8 (3rd order, with constraints)	4957.757	5034.154	0.427

Table 12 shows the forecasted total number of reported cases according to the posterior predicted distribution and the realized values of the proposed discrepancy measures defined in (9) and ${\tilde{\text{Dist}}}_{t}$, along with the out‐of‐fit posterior *p*‐value for each day. We observe that the *p*‐values obtained with the Dirichlet‐multinomial models are higher than those in Table 4 referred to the Italian data, thus showing a better predictive power, probably because the observations collected within the region are more homogeneous than those collected over the entire nation.

**TABLE 12:** Realized values of the discrepancy measures according to Models 7 and 8 for the forecasted cases in Lombardy and posterior *p*‐values over a period of 10 days

	Model 7			Model 8		
Day	${\hat{\text{Dist}}}_{t}$	${\tilde{\text{Dist}}}_{t}$	*p*‐value	${\hat{\text{Dist}}}_{t}$	${\tilde{\text{Dist}}}_{t}$	*p*‐value
25th April	46.720	88.258	0.609	7.588	38.309	0.810
26th April	193.641	150.450	0.368	28.269	72.218	0.641
27th April	739.840	202.970	0.114	177.185	103.660	0.216
28th April	1282.245	249.381	0.076	245.815	132.068	0.229
29th April	2055.032	289.949	0.050	317.009	157.467	0.236
30th April	3136.772	332.528	0.034	409.247	180.781	0.220
1st May	4614.224	375.590	0.024	512.889	204.401	0.202
2nd May	6496.372	421.960	0.019	603.037	226.130	0.184
3rd May	8749.014	472.851	0.015	629.558	246.278	0.201
4th May	12072.930	536.123	0.011	783.759	266.754	0.174

Table 13 shows that the number of H and ICU patients is predicted with minimum error. This confirms that our proposal is particularly appropriate to predict mortality risk as well as progression to severe disease.

**TABLE 13:** Realized values of the discrepancy measure for each category referred to the observed and predicted counts for Lombardy over a period of 10 days

	S	R	Q	H	ICU	D	Total
Model 7 ${\tilde{\text{Dist}}}_{k}^{*}$	1.000	5492	3426	88	14	16	9037
Model 8 ${\tilde{\text{Dist}}}_{k}^{*}$	0.000	272	573	1116	18	10	1990

#### 4.2.2 Results obtained from Model 8 for Lombardy

**FIGURE 3:**
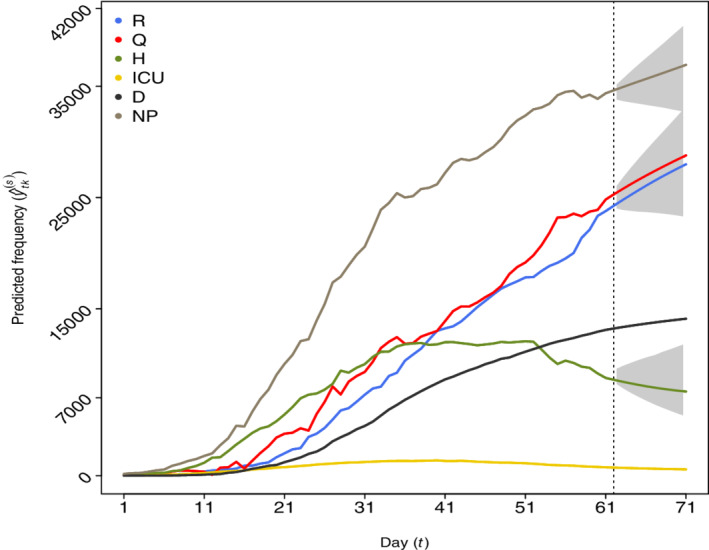
Observed frequencies (before the vertical dashed line corresponding to the 25th of April) and 5 days predictions (after the vertical line until the 4th of May) for categories: recovered (R), positive cases in quarantine (Q), hospitalized (H), intensive care (ICU), deceased (D), and “now positive” (NP). The estimated 95% prediction intervals are visualized in gray [Colour figure can be viewed at wileyonlinelibrary.com]

Figure 3 shows the daily observed and predicted counts with a time horizon of 10 days along with the estimated 95% prediction intervals depicted in gray.

The posterior means of the predicted transition frequencies between categories referred to the first predicted day are shown in Table 14. The estimated 95% prediction upper and lower bounds are reported in Table 15. The estimated posterior mean and the 95% predicted interval for the increase in totals for H and ICU are reported in Table 16.

**TABLE 14:** Estimated posterior means of the predicted transitions between categories with obtained with Model 8 from 25th to 26th of April (from the 61st to the 62nd day)

	S	R	Q	H	ICU	D
**S**	9 988 451	0	774	93	0	0
**R**	0	23 779	3	0	0	0
**Q**	0	309	24 123	389	0	0
**H**	0	170	379	8142	28	72
**ICU**	0	0	0	0	703	52
**D**	0	0	0	0	0	13 106

**TABLE 15:** Estimated posterior 95% prediction upper and lower bounds for the transitions between categories from 25th to 26th of April (from the 61st to the 62nd day)

	S	R	Q	H	ICU	D
**S**	—	(0, 0)	(246, 1210)	(0, 581)	(0, 1)	(0, 0)
**R**	—	(23 759, 23 782)	(0, 22)	(0, 0)	(0, 0)	(0, 0)
**Q**	—	(0, 1337)	(22 668, 24 796)	(0, 1565)	(0, 0)	(0, 0)
**H**	—	(0, 510)	(70, 737)	(7683, 8446)	(4, 67)	(30, 121)
**ICU**	—	(0, 0)	(0, 0)	(0, 5)	(668, 731)	(24, 87)
**D**	—	—	—	—	—	—

**TABLE 16:** Estimated posterior means and 95% prediction intervals (PI) of the increase in totals for H and ICU over a period of 10 days obtained with Model 8

Day	H	PI	ICU	PI
25th April	−167	(−679, 988)	−24	(−61, 18)
26th April	−160	(−672, 1014)	−23	(−58, 16)
27th April	−147	(−656, 1058)	−22	(−54, 15)
28th April	−141	(−645, 1062)	−20	(−51, 15)
29th April	−130	(−635, 1082)	−19	(−48, 14)
30th April	−119	(−609, 1085)	−18	(−45, 14)
1st May	−105	(−596, 1116)	−17	(−43, 13)
2nd May	−94	(−573, 1110)	−15	(−41, 13)
3rd May	−87	(−562, 1090)	−14	(−39, 13)
4th May	−73	(−538, 1095)	−13	(−37, 13)

**FIGURE 4:**
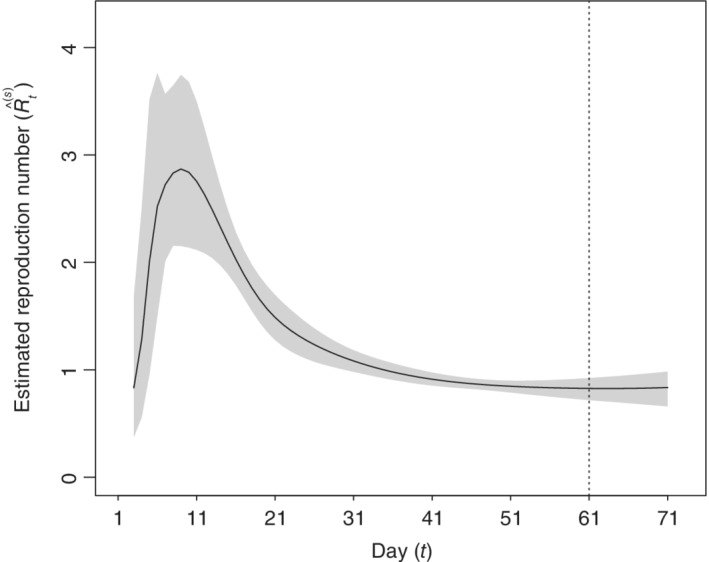
Estimated and predicted (from the vertical line) reproduction number $R_{t}$ for Lombardy obtained with Model 8 (61 observed days, prediction from 25th of April to 4th of May). The estimated 95% credibility and prediction intervals are displayed in gray

Figure 4 displays the dynamics of $R_{t}$ as obtained from the estimated model. We found that, on average, this value increases over time during the early phase of the pandemic. Its decrease begins on the 11th day, corresponding to the 5th of March, a few days after the issuance of the NPIs.

#### 4.2.3 Some results obtained from Model 7 for Lombardy

In the following we show some results obtained adopting Model 7 for the Lombardy region. The posterior means of the predicted transition frequencies referred to the 25th and the 26th of April are reported in Table 17. In this model no constraints are imposed on the odds for the transitions across categories.

The estimated posterior mean and the 95% predicted interval for the increase in totals for H and ICU are reported in Table 18. We notice that the length of the predicted intervals is higher than that estimated with Model 8, and it increases with the number of predicted days.

**TABLE 17:** Estimated posterior predicted transitions between categories from the 25th to the 26th of April (from the 61st to the 62nd day) according to Model 7

	S	R	Q	H	ICU	D
**S**	9 988 204	529	276	295	0	14
**R**	0	23 063	64	652	2	2
**Q**	0	814	22 782	1220	4	2
**H**	0	81	1940	6635	49	86
**ICU**	0	1	0	5	674	76
**D**	0	0	0	0	0	13 106

## 5 DISCUSSION

We propose a novel Bayesian approach based on multinomial and Dirichlet‐multinomial distributions for time‐series counts which can be useful to understand the diffusion of the coronavirus pandemic and to forecast, with good accuracy, for some days ahead, the expected number of people in the following categories: susceptible not previously ill, recovered, positive cases in quarantine, hospitalized, intensive care, and deceased. However, the models are formulated in a general way, and may be adapted to a different number of categories according to data availability. Moreover, they transcend the COVID‐19 context since they can be suitable for many other situations where the assumption on the sequence of contingency tables of the “transition frequencies” between two consecutive time occasions is appropriate. For example, they could be used for the analysis of the transitions between categories of malignant tumors as in the tumor, node, metastasis classification when it is conducted on aggregated data or for the analysis of the transitions between levels of severity of other diseases.

**TABLE 18:** Estimated posterior means and 95% prediction intervals (PI) of the increase in totals for H and ICU over a period of 10 days obtained with Model 7

Day	H	PI	ICU	PI
25th April	15	(−1128, 1778)	−28	(−90, 44)
26th April	62	(−1220, 2017)	−25	(−90, 60)
27th April	113	(−1326, 2224)	−21	(−91, 81)
28th April	155	(−1451, 2451)	−16	(−92, 109)
29th April	180	(−1593, 2644)	−11	(−91, 144)
30th April	214	(−1761, 2924)	−6	(−94, 184)
1st May	221	(−1959, 3109)	2	(−98, 248)
2nd May	252	(−2118, 3426)	12	(−100, 323)
3rd May	270	(−2278, 3676)	21	(−102, 408)
4th May	272	(−2496, 3960)	32	(−103, 524)

The problem of low data quality has been illustrated by Wynants et al.^46^ We recognize that Italian official data may suffer from high variability and may lack representativeness over the population. This might cause an underestimation of the posterior and predictive uncertainties even if the proposed Dirichlet‐multinomial model is also meant to mitigate this issue. We remark that in the present work, we use data provided officially by the national authorities that do not account for the counts of asymptomatic cases. This subcategory of individuals whose infection and recovery both go silent, could not be included in the model, and this constitutes a limitation of the study.

In particular, the proposed prediction models based on a Dirichlet‐multinomial distribution can be used to support medical decisions, especially the management of intensive care units, and to plan increase in critical care bed capacity during the emergency. As stated by Remuzzi and Remuzzi,^47^ the prediction is very important to plan new facilities all over the countries and regions. The early identification of needs is a crucial aspect both for policy makers, and for physicians. Once epidemiological hypotheses are introduced, our model can be interpreted in the same spirit of more standard SEIR models and it can be used to estimate the daily reproduction number. With respect to SEIR models, it is more exploratory because it requires fewer assumptions and hypotheses. Moreover, we do not preclude transitions among the observed categories but we only place minimum requirements in the odds that are knowledge domain driven, which results in more stable estimates.

We are also aware that the pandemic may be seen as many local epidemics that are dependent on each other. Still, we believe that this does not create problems in interpreting our results since we model the joint time series without explicitly dealing with the interactions.

A possible extension of the proposed model would be to consider a set of nodes that correspond to different regions, and a network would describe the flow of people (either infected or susceptible) between nodes. A model having a multinomial or a Dirichlet‐multinomial distribution could be fitted at each node, and interactions between nodes could be explicitly modeled. However, interesting comparisons can be made even within our proposal since the model can be estimated with data at the regional level and then a comparison of transition rates among categories across regions can be performed and the time spent in each category can be compared across regions. This analysis may be useful also to better understand the dynamics of the deceased, which are remarkably different among the Italian regions. We are confident that our proposal may help better plan active public health interventions in the feature and avoid the development of critical illness for patients.

## Data Availability

We use publicly available data and in the Appendix we provide the link to the data source.

## Footnotes

*
https://www.istat.it/it/files//2020/07/Report_ISS_Istat_Inglese.pdf, accessed 3 December 2020.

† Data from the Johns Hopkins Coronavirus Resource Center see https://coronavirus.jhu.edu/about, accessed 3 December 2020.

## 1

### Additional details

The proposed multivariate Bayesian statistical approach relies on a Markov chain Monte Carlo (MCMC) algorithm based on two steps which are iteratively repeated as described in Section 3.1. Regarding the first step, which consists in updating the transition tables, we propose a possible switch that consists in adding (or subtracting) a random integer number between 1 and 50 to the cells in the main diagonal of a randomly selected 2×2 subtable, and subtracting (or adding) the same number to the off‐diagonal cells. Regarding the second step, which consists in drawing new values for all vectors of regression parameters $\beta_{jk}$, we use a multivariate normal proposal distribution with mean centered on the current vector and variance‐covariance matrix equal to $0.3^{2}I$, even if the algorithm that we make available allows the user do adopt a different proposal variance for each *j* and *k*. The MCMC algorithm is run for 500 000 iterations in addition to the initial 100 000 iterations considered as burn‐in. The thinning parameter is set to 10 in order to reduce the autocorrelation along the path of the Markov chain.

The MCMC algorithm, run with the data of the illustrated applications on a desktop computer with 2.7‐GHz Intel(R) Core(TM) i7 quad core processor, requires a computational time of approximately 7 hours.

In order to assess the performance of the MCMC algorithm, we have to consider that the proposed model based on the Dirichlet‐multinomial distribution, so as to include overdispersion, is essentially overparametrized with respect to the number of observations. However, as already illustrated in Section 2.2, we recover numerical stability thanks to the adoption of inequality constraints on the odds. Moreover, we notice that the algorithm performs adequately in terms of predictions, which represent a crucial aspect in applications of this type. We note that these predictions are convolutions of the frequencies contained in the forecasted transition tables. In this regard, we show in Table A1 the algorithm's effective sample size for the forecasted counts in the various categories of interest (S, R, Q, H, ICU, D) for 1, 2, and 3 days ahead obtained with Models 7 and 8 using data for Italy and Lombardy. In updating the regression parameters, the acceptance rate is on average 26% for model 8 estimated with the Italian data, and this is in line with the literature.^48^ For each case, the effective sample size is calculated as the ratio between the number of available MCMC replications, 50 000, and the integrated autocorrelation time obtained by the IAT function of the R library LaplacesDemon.

**TABLE A1:** Effective sample sizes of the estimated posterior means of the predicted counts in the various categories of interest for 1, 2, and 3 days ahead obtained with Models 8 and 7 for the Italian and Lombardy data

	Italian data						Lombady data					
	Model 8			Model 7			Model 8			Model 7		
	Day 1	Day 2	Day 3	Day 1	Day 2	Day 3	Day 1	Day 2	Day 3	Day 1	Day 2	Day 3
**S**	12 893	5641	3677	6911	2049	897	15 297	6264	4747	11 042	5185	2034
**R**	11 605	4611	2865	4768	2129	1603	22 411	12 527	7016	12 561	2236	1844
**Q**	12 257	4288	3672	4660	2731	1046	10 335	5408	3340	12 936	2568	1878
**H**	20 548	3968	2892	3928	2459	1546	14 440	11 382	8385	12 907	3688	1779
**ICU**	16 892	4067	2914	14 014	3280	1767	31 998	23 413	19 022	10 947	5219	3195
**D**	16 512	6712	3447	3757	2463	1538	42 671	31 295	31 413	13 507	6212	2481

A final note concerns the data source. To define the number of the susceptible individuals, we used the last population census data provided by the Italian Statistical Institute (available at the following link https://www.istat.it/it/archivio/238447, accessed 3 December 2020). Daily counts on R, Q, H, ICU, D can be downloaded from the following repository https://github.com/pcm‐dpc/COVID‐19/. For reproducibility purposes, the code to estimate the proposed models written for the open source software R ^49^ is available from the Github repository at the following link https://github.com/francescobartolucci/ARMultinomial.
